# Oncologic and functional outcomes of different reconstruction modalities after resection of chondrosarcoma of the scapula: a medium- to long-term follow-up study

**DOI:** 10.1186/s12891-022-05661-7

**Published:** 2022-08-08

**Authors:** Xiao-Jun Yu, Qi-Kun Liu, Ying-Guang Wang, Shan-Xi Wang, Rui Lu, Hao-Ran Xu, Jun-Lai Wan, Hao Kang

**Affiliations:** Department of Orthopedics, Tongji Hospital, Tongji Medical College, Huazhong University of Science and TechnologyQiaokou DistrictHubei Province, No. 1095, Jiefang Avenue, 430030 Wuhan, China

**Keywords:** Scapula, Chondrosarcoma, Scapulectomy, Prosthesis, Reconstruction

## Abstract

**Objectives:**

To evaluate the oncologic and functional results of scapular reconstruction after partial or total scapulectomy for chondrosarcoma.

**Materials and methods:**

Twenty-one patients with chondrosarcoma who underwent partial or total scapulectomy between January 2005 and July 2019 were reviewed retrospectively.

**Results:**

At a mean follow-up of 62.6 months (range, 13–123 months), four patients developed local recurrence, and three developed distant metastases, one of which developed both recurrence and metastasis. The overall survival rate of patients at 5 years was 84.6%, the disease-free survival rate was 69.3%, and the complication rate was 19% (4/21). The 1993 American Musculoskeletal Tumor Society (MSTS93) scores of patients in the partial scapulectomy group, total scapulectomy + humeral suspension group and prosthetic reconstruction group were 26.50 ± 1.38, 19.00 ± 2.58, and 21.38 ± 2.62, respectively. There was a statistically significant difference between the partial scapulectomy group and the total scapulectomy + humeral suspension or prosthetic reconstruction group ( *P* = 0.006 and 0.0336, respectively). The range of motion of the shoulder joint for forward flexion was 80.83° ± 11.14°, 51.25° ± 21.36°, and 52.50° ± 11.02°, respectively. The p-values for the comparison between the partial scapulectomy group and the total scapulectomy + humeral suspension or prosthetic reconstruction group were 0.0493 and 0.0174, respectively. And the range of motion of abduction was 75.00° ± 10.49°, 32.50° ± 11.90°, 41.88° ± 11.63°, respectively. Patients in the partial scapulectomy group had significantly better postoperative shoulder abduction function than the total scapulectomy + humeral suspension or prosthetic reconstruction group (*P* = 0.0035 and 0.0304, respectively). There was no significant difference in MSTS93 scores and flexion and abduction function of the shoulder joint in the upper extremity after total scapulectomy with humeral suspension or prosthetic reconstruction (*P* > 0.05).

**Conclusions:**

Surgical treatment of chondrosarcoma of the scapula can achieve a satisfactory prognosis and shoulder function. Total scapulectomy followed by prosthetic reconstruction or humeral suspension are both feasible treatments.

**Supplementary Information:**

The online version contains supplementary material available at 10.1186/s12891-022-05661-7.

## Highlights

Surgical treatment of chondrosarcoma of the scapula can achieve good oncologic and functional outcomes.

Prosthetic reconstruction of the scapula after scapulectomy does not provide better functional results than humeral suspension, and both are feasible treatment modalities.

**Supplementary Information:**

The online version contains supplementary material available at 10.1186/s12891-022-05661-7.

## Introduction

Chondrosarcoma occurring in the scapula is rare. Tumors occurring in the scapula and other flat bones have shown worse prognoses than those occurring in the extremities [1, 2]. Meanwhile, chondrosarcoma is not sensitive to radiotherapy or chemotherapy; thus, to better control the development of the tumor, patients with malignant tumors occurring in the scapula have previously required amputation [3, 4]. With the advancement of neoadjuvant therapy, surgical technology and materials, limb salvage therapy for malignant tumors of the scapula has reached approximately 95% [5, 6]. Current studies have shown that limb salvage treatment has comparable or even better outcomes than amputation [7]. However, reconstruction after scapulectomy has been an important challenge for clinicians because of the special location of the scapula adjacent to important neurovascular and cardiopulmonary structures. To date, there are three main reconstruction strategies. One is humeral suspension treatment, in which the rotator cuff or joint capsule is directly sutured or reconstructed with the proximal structures, such as the clavicle or the residual scapula. The second is reconstruction with autologous or allogeneic bone grafts, and the third is the more prevalent regimen, in which reconstruction is performed with a constrained or nonconstrained prosthesis. These different reconstruction modalities are reported to have their own advantages and corresponding complications [2, 8–12]. To date, most of the studies on reconstruction after resection of malignant tumors of the scapula have included small sample sizes and short- to medium-term follow-up results, and there is a lack of studies comparing the functional outcomes of different reconstruction modalities. In addition, few studies have reported the outcome of scapula resection and reconstruction for chondrosarcoma. Our aim was to review the cases of scapular chondrosarcoma with scapulectomy in our hospital; summarize the postoperative tumor recurrences, metastasis and postoperative complications; and evaluate the functional outcome of the upper limb after surgery by the 1993 American Musculoskeletal Tumor Society (MSTS93) scores. Additionally, we compared the results of different scapular reconstruction modalities.

## Materials and methods

### Patient inclusion criteria

(1) Chondrosarcoma originating from the scapula or invading the scapula from the surrounding soft tissues; (2) Postoperative positive pathological diagnosis of chondrosarcoma; (3) Partial or total scapulectomy + scapular reconstruction; (4) Enneking surgical staging of no more than stage IIB.

### Exclusion criteria

(1) No definite histological diagnosis; (2) The tumor was located in the soft tissue around the scapula without invasion of bone; (3) No reconstruction after scapulectomy.

### Surgical procedures

**Fig. 1:**
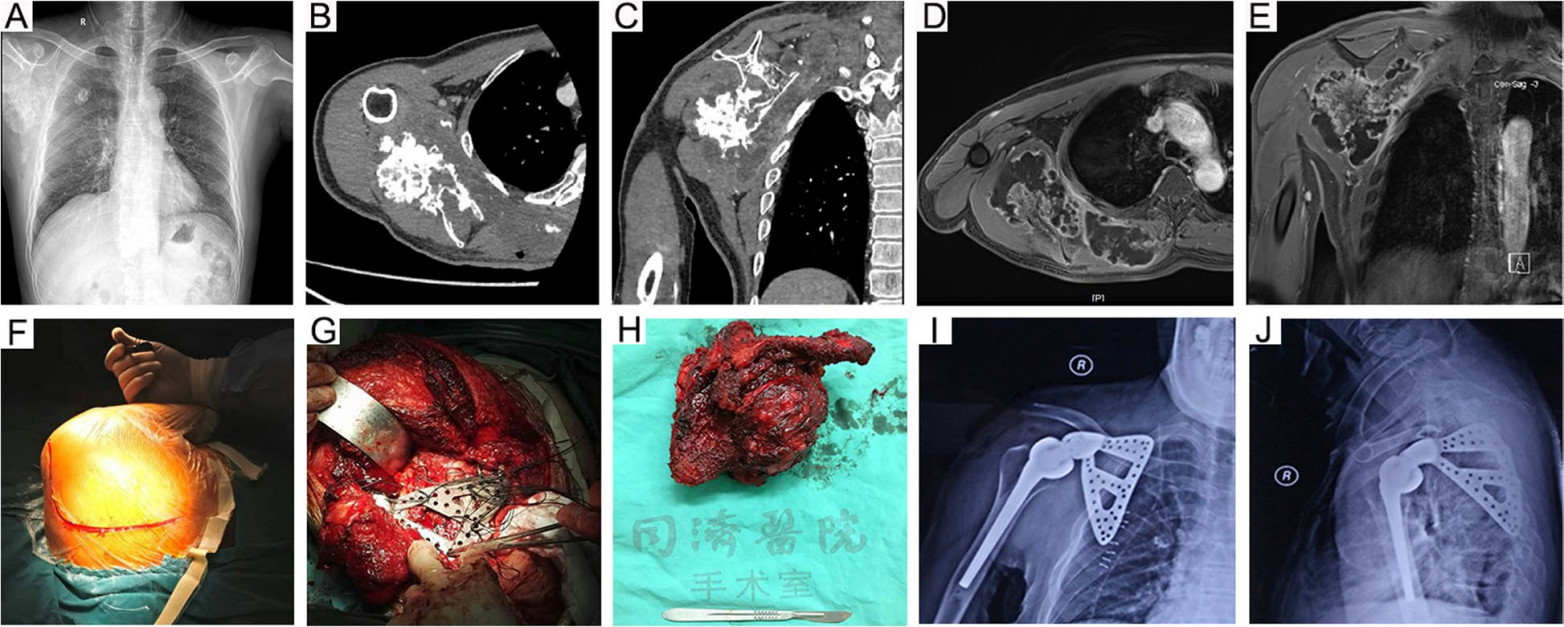
A 58-year-old male patient with right scapular common chondrosarcoma underwent total scapulectomy + prosthesis for common chondrosarcoma of the right scapula (case #5 in Table 1). **A**: Preoperative plain radiograph. **B**-**D**: Cross-sectional and coronal CT imaging. **D**-**E**: Magnetic resonance imaging (T2WI cross-section and coronal). **F**: Surgical incisional approach. **G**: Soft tissue reconstruction around the prosthesis. **H**: Excised specimens. **I**-**J**: Postoperative anteroposterior and lateral plain radiographs

**Fig. 2:**
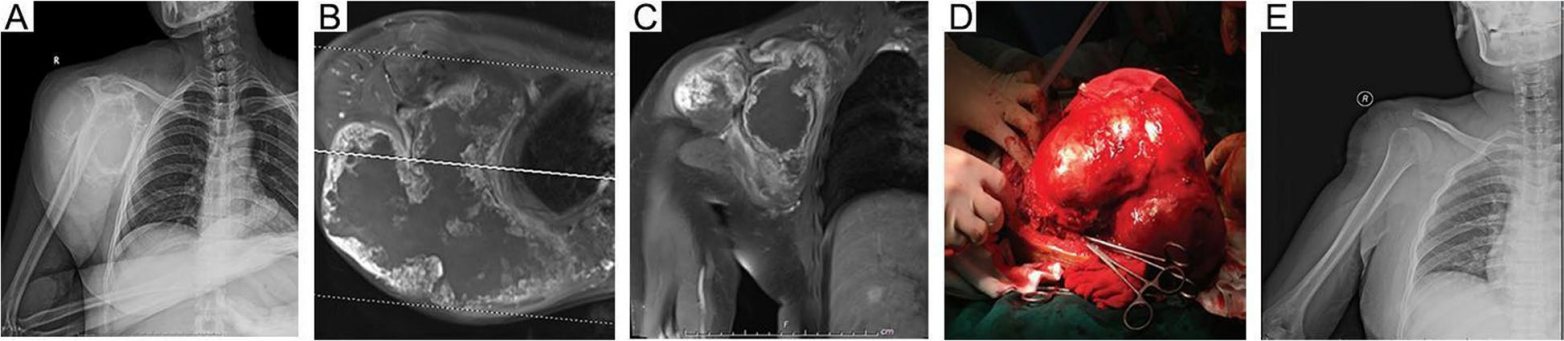
A 49-year-old female patient underwent total scapulectomy + humeral suspension for giant highly differentiated chondrosarcoma of the right scapula (case #4 in Table 1). **A**: X-ray imaging showing osteolytic destruction of the right scapula. **B**-**C**: MRI showed a massive tumor mass in the right shoulder (T1WI). **D**: Images of intraoperative tumors. **E**: Postoperative radiography showed that the shoulder was in good shape

**Fig. 3:**
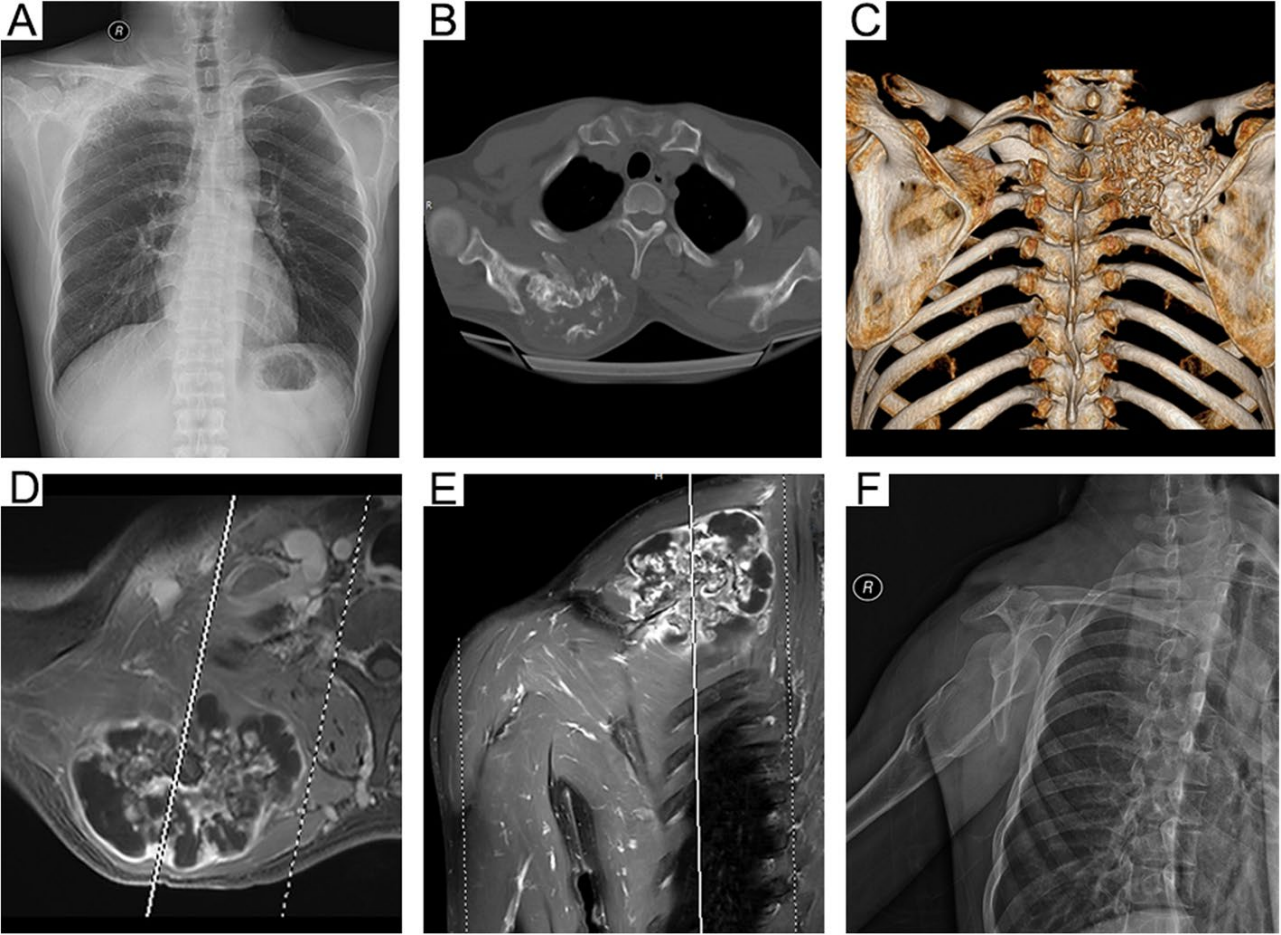
A 27-year-old male with right scapular common chondrosarcoma due to malignant transformation of hereditary multiple osteochondromas (case #6 in Table 1). **A**: Preoperative anteroposterior plain radiograph. **B**-**C**: Cross-sectional CT scan and three-dimensional reconstruction imaging. **D**-**E**: Magnetic resonance imaging (T1WI cross-section and coronal). **F**: Postoperative anteroposterior X-radiograph imaging

After anesthesia, the patient was placed in the lateral supine or lateral prone position with the ipsilateral shoulder up. Then, through a combined anterior and posterior surgical approach, the incision began from the coracoid process, crossed the outer 1/3 of the clavicle, followed the spine of the scapula and the medial border of the scapula to the inferior angle of the scapula, and sequentially incised the tissue structures to the tumor lesion (Fig. 1F). The extent of surgical resection was determined by the tumor border shown by preoperative MRI, and the normal tissue structures 2–3 cm around the tumor lesion were removed together. According to the location and extent of the scapular tumor, surgical resection and reconstruction were classified into three types. Partial scapuletomy was defined as partial resection of the scapula with preservation of the glenoid, and resection of either the supraspinous, infraspinous or acromion. Total scapulectomy was defined as resection of the glenoid of the shoulder together. In our series, 10 patients were treated with prosthetic reconstruction after total scapulectomy (Fig. 1, prostheses are all from Chunli Zhengda Medical Company of Beijing, China), 5 with humeral suspension after total scapulectomy (Fig. 2), and 6 with partial scapulectomy (Fig. 3). All patients underwent soft tissue reconstruction after scapulectomy. The biceps was sutured to the clavicle, the deltoid was sutured to the trapezius, the serratus anterior was sutured to the latissimus dorsi and rhomboid, and the soft tissue surrounding the prosthesis could be sutured directly to the anchoring holes at the edge of the prosthesis by nonabsorbable sutures (Fig. 1G). The main purpose was to restore the structural integrity and stabilizing role of the deltoid, biceps, and rotator cuff. Finally, the drainage tube was placed, and the incision was closed layer by layer.

### Postoperative patient management and follow-up

After surgery, patients received fluid administration and antibiotic therapy to prevent wound infection. The drainage tube is removed when the volume of drainage fluid is less than 30 ml/d, generally for 2–3 days. Postoperatively, the patient wore a brace to maintain the affected limb at 90° elbow joint flexion, 60° shoulder joint flexion and 60° abduction, and the brace was fixed for 4–6 weeks. Patients were encouraged to exercise the hands, wrists, and elbows early after surgery and gradually exercise the shoulder joints after the brace was removed.

After discharge from the hospital, regular outpatient follow-up was performed once a month within 3 months, once every 3 months within 3–12 months, once every 6 months for 1–3 years, and then once a year. X-ray examination of the shoulder was performed during each outpatient follow-up to assess whether the tumor had recurred, whether the prosthesis was loose and other abnormalities. For suspicious cases, CT or MRI tests were performed for further confirmation. A CT scan of the lungs was performed every six months to one year to exclude metastases. At the final follow-up, the MSTS93 scoring system was used to evaluate the patient's shoulder function, including pain, function, emotional acceptance, hand position, manual dexterity, and lifting ability. Each item was 0–5 points, with a total score of 30 points.

### Statistical analysis

GraphPad Prism 8.0 (Graphpad Inc, San Diego, CA, USA) software was used for data processing and statistical analysis. Overall survival and disease-free survival were estimated using the methods of Kaplan and Meier. The results of continuous variables are shown as the means and standard deviation. The functional results between the three groups were compared by the nonparametric Kruskal–Wallis test because these data were not normally distributed.

## Results

From January 2005 to July 2019, 21 eligible patients were treated in our hospital. Among them, 13 were males, and 8 were females. The patients were 27–65 years old, with an average age of 47.5 years. All patients underwent surgical treatment, of which 15 patients achieved wide surgical margins, and 6 underwent intralesional or marginal resection because of tumor invasion of the ribs, chest wall, or adjacent important neurovascular structures. All cases were pathologically confirmed as chondrosarcoma, most of which were conventional type (13/21). The complication rate was 19% (4/21), with no serious complications occurring after surgery. Two patients had wound healing problems (cases 4 and 18), which were completely healed after long-term dressing change and anti-infection treatment for approximately 1 month. One patient developed postoperative upper limb paresthesia, which recovered after neurotrophic treatment at approximately 3 months postoperatively. Shoulder dislocation occurred in one patient, and function was not significantly affected after open reduction. No patient had complications such as fracture, skin flap necrosis, deep soft tissue infection, or prosthesis loosening.

**Fig. 4:**
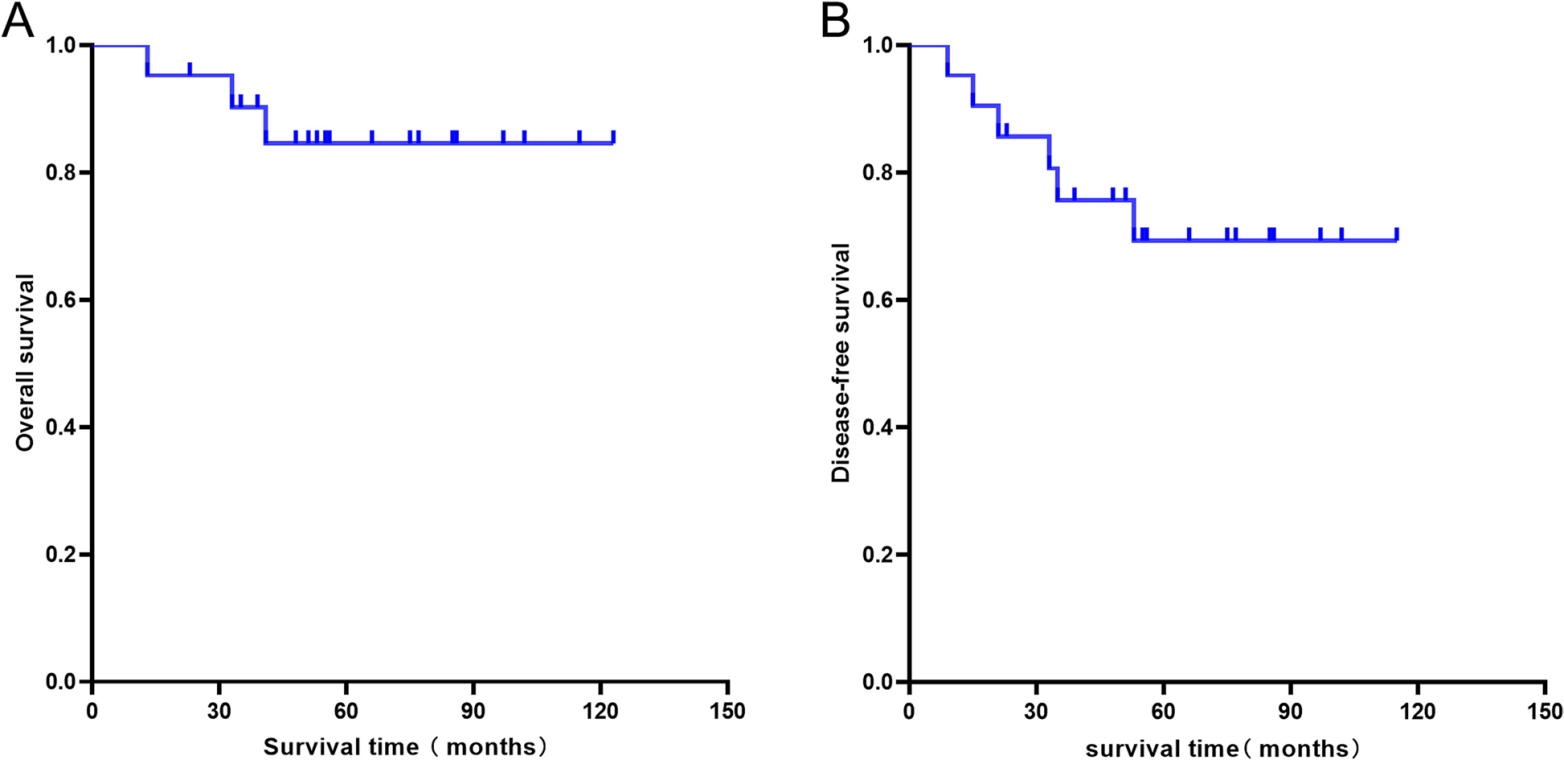
Kaplan–Meier curve showing the overall survival rate and disease-free survival rate of 21 patients

As shown in Table 1, at a mean follow-up of 62.6 months (range, 13–123 months), eighteen patients were still alive at the final follow-up, three of whom survived with disease. Of the remaining 3 patients, 2 died of systemic multiple organ failure caused by lung metastasis at 13 and 41 months after surgery, respectively, and 1 patient died of cardiac disease at 33 months postoperatively (case 20). Among all patients, four had local recurrence after surgery, and three had distant metastases, one of whom had both recurrence and metastasis (case 5). The overall survival rate of patients at 5 years was 84.6%, and the disease-free survival rate was 69.3%. The Kaplan–Meier survival curve is shown in Fig. 4.

**Table 1:** Patient characteristics, surgical modalities and outcomes (2005/1–2019/7)

Case	Gender/Age(years)	Histological types	Enneking Stage	Malawer classification	Surgical modality	Margin	Complications	Relapse/metastasis	Status	Follow-up(months)
1	M/34	Mesenchymal	IIB	IIA	Partial scapulectomy	Marginal	No	Relapse	AWD	123
2	F/56	Conventional	IB	IIA	Partial scapulectomy	Wide	No	No	NED	75
3	M/46	Myxoid	IIA	IIIA	Total resection + humeral suspension	Wide	No	Relapse	NED	85
4#	F/49	Conventional	IB	IIIA	Total resection + humeral suspension	Marginal	Poor wound healing	No	NED	56
5#	M/58	Conventional	IIB	IIIA	Total resection + Prosthesis	Intralesional	No	Relapse + Metastasis	AWD	41
6#	M/27	Conventional	IB	IIA	Partial scapulectomy	Wide	No	No	NED	23
7	M/58	Myxoid	IIB	IIIA	Total resection + Prosthesis	Wide	No	No	NED	86
8	M/32	Conventional	IIB	IIIA	Total resection + Prosthesis	Wide	Dislocation	No	NED	102
9	M/48	Dedifferentiated	IIB	IIIA	Total resection + Prosthesis	Wide	No	Metastasis	DOD	13
10	F/50	Myxoid	IIB	IIIA	Total resection + Prosthesis	Wide	No	Relapse	AWD	35
11	F/65	Dedifferentiated	IIB	IIIA	Total resection + Prosthesis	Wide	No	No	NED	53
12	M/35	Mesenchymal	IIB	IVA	Total resection + humeral suspension	Marginal	No	No	NED	115
13	F/48	Conventional	IIB	IVA	Partial scapulectomy	Wide	No	No	NED	97
14	M/58	Conventional	IIB	IVA	Total resection + humeral suspension	Wide	No	Metastasis	DOD	41
15	M/59	Myxoid	IIB	IVA	Total resection + humeral suspension	Intralesional	Paresthesia	No	NED	77
16	F/49	Conventional	IB	IIIA	Total resection + Prosthesis	Wide	No	No	NED	66
17	M/50	Conventional	IIB	IIA	Partial scapulectomy	Wide	No	No	NED	55
18	F/41	Conventional	IIB	IIIA	Total resection + Prosthesis	Wide	Wound necrosis	No	NED	48
19	M/27	Conventional	IB	IIIA	Total resection + Prosthesis	Wide	No	No	NED	39
20	M/65	Conventional	IIB	IIIA	Total resection + Prosthesis	Marginal	No	No	DOD	33
21	F/42	Conventional	IIB	IIA	Partial scapulectomy	Wide	No	No	NED	51

*M* male, *F* Female, *AWD* Alive with disease, *NED* No evidence of disease, *DOD* Died of disease, #, representative case.

**Fig. 5:**
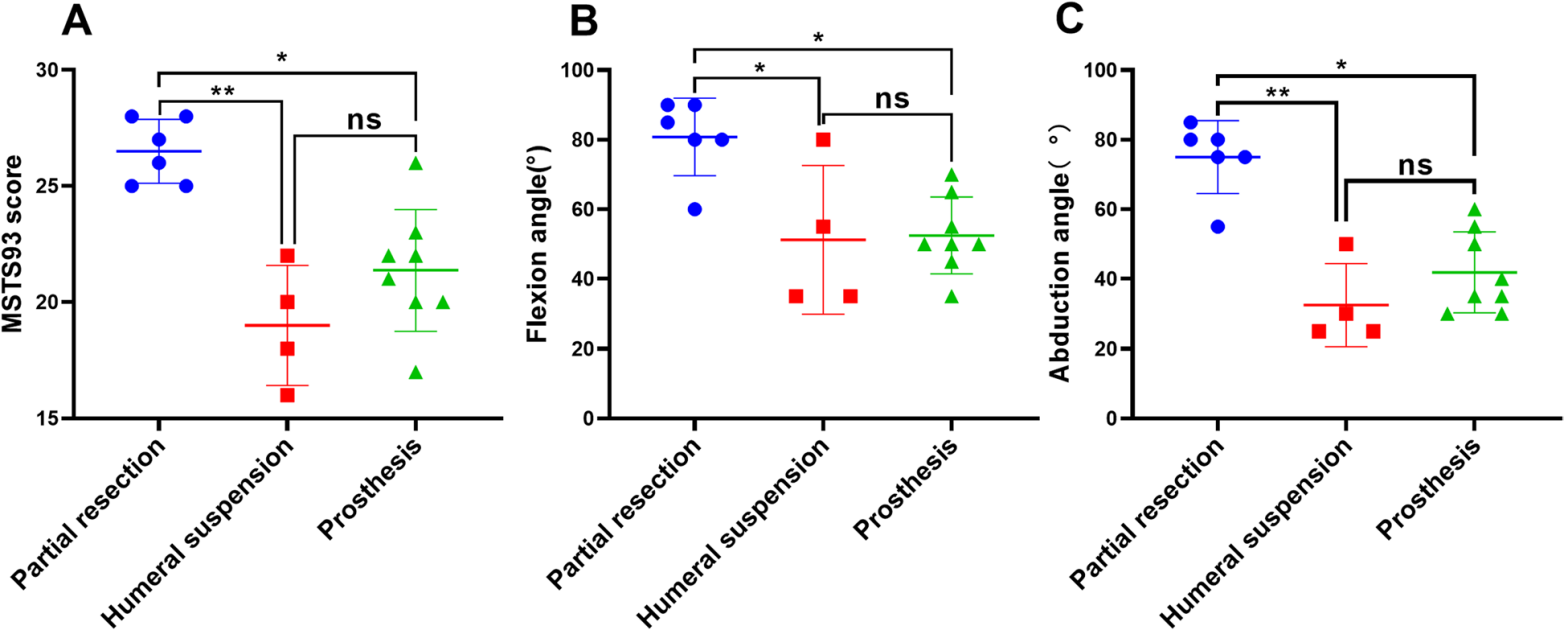
MSTS93 scores, range of motion in forward flexion and abduction of the shoulder joint and comparative results of patients with chondrosarcoma of the scapula among three different treatment groups. *, *p* value < 0.05; **, *p* value < 0.01

As shown in Table 2, we evaluated postoperative shoulder function and upper extremity MSTS93 scores in 18 patients. Six patients who underwent partial scapulectomy had significantly better shoulder function, with MSTS93 scores, shoulder forward flexion and abduction range of 26.50 ± 1.38, 80.83° ± 11.14°, and 75.00° ± 10.49°, respectively. We further compared the postoperative shoulder function in the three groups and the results are shown in Additional file 1: Supplementary Table 1 and Fig. 5. The partial scapulectomy group compared with the total scapulectomy + humeral suspension or prosthesis reconstruction group had better MSTS93 scores (*P* = 0.006 and 0.0336, respectively), larger range of motion for shoulder flexion (*P* = 0.0493 and 0.0174, respectively) and abduction (*P* = 0.0035 and 0.0304, respectively). However, there was no significant difference in postoperative MSTS93 scores and shoulder range of motion between patients in the humeral suspension and prosthetic reconstruction groups (*P* > 0.05). Moreover, most patients achieved good pain control and satisfactory shoulder contours, and all patients maintained normal hand, wrist and elbow function postoperatively.

**Table 2:** Functional scores and range of motion of the shoulder joint after resection of chondrosarcoma of the scapula with different reconstruction modalities

Resection type	Case	MSTS93 score	Flexion	Abduction
Partial resection	1	27	85°	80°
	2	25	60°	55°
	6	26	90°	85°
	13	28	90°	80°
	17	28	80°	75°
	21	25	80°	75°
Mean ± SD	/	26.50 ± 1.38	80.83° ± 11.14°	75.00° ± 10.49°
Humeral suspension	3	18	55°	30°
	4	22	80°	50°
	12	16	35°	25°
	15	20	35°	25°
Mean ± SD	/	19.00 ± 2.58	51.25° ± 21.36°	32.50° ± 11.90°
Prosthesis reconstruction	5	22	50°	30°
	7	20	50°	35°
	8	23	65°	50°
	10	20	45°	40°
	11	22	55°	35°
	16	26	50°	55°
	18	17	35°	30°
	19	21	70°	60°
Mean ± SD	/	21.38 ± 2.62	52.50° ± 11.02°	41.88° ± 11.63°

*SD* Standard deviation

## Discussion

Before the 1970s, shoulder disarticulation was the main treatment for malignant bone tumors of the shoulder girdle [13], and the appearance and function of the affected limb were seriously damaged, placing a serious burden on the psychological and social functions of the patients. In 1857, Syme [14] first reported total scapulectomy for the treatment of malignant scapular tumors. In 1999, Nakamura et al. [15] showed that patients who undergo total scapulectomy may achieve much better upper limb function than those who undergo forequarter amputation. Subsequently, an increasing number of patients with scapular malignant tumors have achieved limb salvage. At present, limb salvage surgery has become the preferred treatment for scapular malignant tumors, and limb salvage treatment can be achieved in approximately 95% of patients [6].

Reconstruction after scapular tumor resection is a major surgical challenge and also has important implications for postoperative shoulder function. Humeral suspension was the most popular reconstruction method after scapulectomy in the early 1990s [12]. A study [16] from Japan included 23 patients who underwent humeral suspension reconstruction, and after a mean follow-up of 61.9 months, the mean Enneking functional score was 21.1 (70.3%), and the active shoulder range of motion was 42.7 degrees in flexion and 39.7 degrees in abduction. The authors concluded that humerus suspension after scapular resection can achieve ideal shoulder function. Xu et al. [17] reported that the average MSTS score of 8 patients with humeral suspension reconstruction was 16.3 (57%), there was no recurrence or major complications after the operation, and the average emotional acceptance was 3.6 (72.5%). However, some studies have found that patients with humeral suspension have problems, such as floating humerus, poor cosmetic outcomes, and restricted joint functions [12], and some patients may experience limb numbness and muscle atrophy due to traction of vascular nerve bundles. In our study, the MSTS93 scores of the four patients who underwent humeral suspension were 19.00 ± 2.58. Of these, two patients had superior shoulder motion with 55° and 80° of forward flexion, and both had more than 30 degrees of abduction. All patients obtained satisfactory shoulder contour and pain control, and no patients had glenohumeral joint droop or flail shoulder (Fig. 2E).

With the development of prosthetic materials, manufacturing processes and surgical techniques, surgeons have made unremitting efforts and attempts to reconstruct the scapular region after tumor resection. In 1987, Eckardt [18] made the first attempt to perform prosthetic reconstruction after total scapulectomy. Since then, several studies have reported that prosthetic reconstruction can achieve good oncological and functional outcomes [8, 19, 20]. Li [21] reported 17 cases of scapular malignant tumors undergoing total scapulectomy and reconstruction with prostheses. After an average follow-up of 45.4 months, the upper limb MSTS function score was 26.1 ± 1.4, and the ranges of shoulder joint flexion, extension, and abduction were 70° ± 7.5°, 31.2° ± 11.3°, and 54.4° ± 12.5°, respectively. The overall postoperative survival rate was 88.2% (15/17), and the disease-free survival rate was 70.6% (12/17). In addition, several studies have shown that prosthetic reconstruction can obtain better shoulder joint function and shape than humeral suspension [22, 23]. Pritsch et al. [12] included 32 patients who had total scapulectomies (reconstructions with humeral suspensions in 16 patients and scapular endoprostheses in 16 patients), and the results showed that scapular endoprosthetic reconstruction led to better functional and cosmetic results than humeral suspension. The mean MSTS scores for patients with scapular endoprostheses and humeral suspensions were 78.5% and 58.5%, respectively. Seven patients with scapular endoprostheses had greater than 40 degrees of abduction, and 11 patients with humeral suspensions could not abduct the shoulder greater than 20 degrees. However, our study found that humeral suspension or prosthetic reconstruction after total scapulectomy both achieved good functional outcomes, with MSTS93 scores of 19.00 ± 2.58 and 21.38 ± 2.62, respectively. Both treatment strategies achieved good shoulder contour and pain control. We did not find significant differences in MSTS93 score and shoulder flexion and abduction function between the two groups, which may be related to the fact that only four cases were treated with humeral suspension, two of which had superior function. Therefore, these cases may not truly reflect the efficacy of humeral suspension. Nevertheless, we found that the patients treated with humeral suspension had poorer shoulder abduction, with only one case beyond 30 degrees.

In addition, the amount of muscles and ligaments preserved around the scapula, the quality of soft tissue reconstruction, and the preservation of the glenoid and acromion have an important impact on improving shoulder function and reducing complications. A study from Japan [16] found that preserving the glenoid or acromion compared to total scapulectomy can achieve better function. Min et al. [20] showed that patients with rotator cuff reconstruction could achieve better upper limb lifting ability and shoulder abduction. Similar studies [24–26] have also reported the importance of the deltoid, subscapular and latissimus dorsi for shoulder function after scapular resection. Our study also demonstrated that a smaller extent of resection of the scapula led to better postsurgical function. Partial scapulectomy had nearly normal shoulder function, which we believe was due to the retention of the acromion and glenoid in these patients. In addition, adequate reconstruction of soft tissues, especially the deltoid, biceps and rotator cuff, is guaranteed to obtain good function of the shoulder joint.

Our study also has several limitations. The first is the relatively small number of cases included in this study, which may have affected the reliability of the conclusions and a larger sample may be required for further validation. Second, this study was retrospective, and the results may have been somewhat biased. Furthermore, the MSTS scores may have been influenced by subjective factors and suffered from insufficient accuracy. Nevertheless, our study is a relatively large series on chondrosarcoma of the scapula, and our results can be helpful for its management.

## Conclusions

Surgical treatment of chondrosarcoma of the scapula can obtain satisfactory oncologic and functional outcomes, and preservation of the important structures of the scapula and adequate reconstruction of the soft tissues are critical to the patient's function. Total scapulectomy followed by prosthetic reconstruction or humeral suspension are both feasible options.

## Supplementary Information

**Additional file 1: Supplementary Table 1**. Comparison of postoperative shoulder function among patients in different treatment groups.

## Data Availability

The datasets generated and/or analyzed during the current study are not publicly available due to limitations of ethical approval involving the patient data and anonymity but are available from the corresponding author on reasonable request.

## Abbreviation

MSTS93: The 1993 American Musculoskeletal Tumor Society
